# The Role of Circulating Tight Junction Proteins in Evaluating Blood Brain Barrier Disruption following Intracranial Hemorrhage

**DOI:** 10.1155/2015/860120

**Published:** 2015-10-25

**Authors:** Xiaoyang Jiao, Ping He, Yazhen Li, Zhicheng Fan, Mengya Si, Qingdong Xie, Xiaolan Chang, Dongyang Huang

**Affiliations:** ^1^Department of Cell Biology and Genetics, Shantou University Medical College, Guangdong 515041, China; ^2^Shantou University Medical College, Guangdong 515041, China

## Abstract

Brain injury after intracranial hemorrhage (ICH) results in significant morbidity and mortality. Blood brain barrier (BBB) disruption is a hallmark of ICH-induced brain injury; however, data mirroring BBB disruption in human ICH are scarce. The aim of this study was to assess the significance of circulating biomarkers in evaluating BBB disruption after ICH. Twenty-two patients with ICH were recruited in this study. Concentrations of the tight junction proteins (TJs) Claudin-5 (CLDN5), Occludin (OCLN), and zonula occludens 1 (ZO-1) and vascular endothelial growth factor (VEGF) and matrix metalloproteinase-9 (MMP-9) were measured by using enzyme-linked immunosorbent assay in serum and cerebrospinal fluid (CSF) samples obtained from patients with ICH. The white blood cell (WBC) count in blood and CSF, albumin (ALB) levels in the CSF (ALB_CSF_), and the BBB ratio were significantly higher in the ICH than in controls (*p* < 0.05). Significantly higher levels of CLDN5, OCLN, ZO-1, MMP-9, and VEGF in CSF were observed in the ICH group; these biomarkers were also positively associated with BBB ratio (*p* < 0.05). Our data revealed that circulating TJs could be considered the potential biomarkers reflecting the integrity of the BBB in ICH.

## 1. Introduction

Spontaneous intracranial hemorrhage (ICH) results in significant morbidity and mortality, thirty-day case fatality rates which range from 40% to 50% in most studies [1–3]. Hypertension is the most important risk factor for ICH [4], where clinical treatment options remain limited, in part, due to the poorly defined sequelae underlying injury progression. Brain injury occurs in the acute injury phase following ICH [5, 6]. BBB disruption is a hallmark of ICH-induced brain injury, which contributes to edema formation, the influx of leukocytes, and the entry of potentially neuroactive agents into the perihematomal brain [7–9]. The degree of BBB breakdown has been directly correlated with late functional outcome [10].

The BBB inhibits transcellular or paracellular passage of molecules across it by an elaborate network of complex tight junctions between the endothelial cells [11]. Tight junction proteins (TJs) are the main components of the BBB [12]. Claudin-5 (CLDN5), ZO-1, and Occludin (OCLN) are the main components of TJs in brain endothelial cells to maintain the BBB integrity. CLDN5 knockout mice presented increased BBB permeability [13]; on the contrary, inhibiting decreased expression of CLDN5 has been shown to reduce brain edema and hemorrhagic transformation [14]. The expressions of ZO-1 and OCLN were significantly decreased after subarachnoid hemorrhage (SAH) [15], and degrading ZO-1 and OCLN in endothelial tight junction can facilitate capillary leakage, which is responsible for the increase in BBB permeability after SAH [16]. Increased BBB permeability after ICH has been noted in parallel with edema formation and BBB disruption leading to vasogenic edema [17]. Presence of TJs in the neurovascular unit is one likely component of the brain's armamentarium against hemorrhage; however, the role of TJs in mirroring the BBB disruption in human ICH is scarce.

The proteins released from damaged cells into the bloodstream would reveal the BBB disruption, which might be potential biomarkers. Protein S100B appears in plasma after ICH and its levels correlated with ICH outcome [18]. Albumin is currently the conventional biomarker used in clinical practice to assess the integrity of BBB. The quotient of CSF/serum albumin (also called BBB ratio) is determined by the concentration of albumin in blood and CSF, which have been used extensively to mirror the BBB disruption in neuroinflammatory disease or in tumor CNS metastasis [19]. However, serum concentrations of S100B and albumin are influenced significantly by status of peripheral circulation and blood released from the hematoma after ICH, leading to the potential inaccuracies in judging the disease severity.

Biomarkers reflecting the severity of ICH could increase the discriminative power for outcome evaluation. Two areas of extensive research are neuroimaging and circulating biomarkers. Circulating TJs are currently becoming the hallmark of BBB integrity [20]. A recent study revealed that the release of TJs into the circulation is expected to occur during brain ischemia; higher serum concentrations of TJs OCLN and CLDN5/ZO-1 ratio were observed in ischemic stroke patients. Analyzing serum TJs is an effective way to screen clinical deterioration caused by hemorrhagic transformation in ischemic stroke patients [21]. Although the mechanism underlying the BBB disruption in ICH remains unclear, inflammatory mediators (such as matrix metalloproteinases (MMPs) and vascular endothelial growth factor (VEGF)) are the crucial factors degrading the TJs. Elevated MMPs and VEGF increased BBB permeability and worsened brain edema after ICH [22–24]. Thus, TJs, degraded by MMPs and VEGF, may be released into CSF after ICH.

Computed tomography (CT) and magnetic resonance imaging (MRI) are the key part of the initial diagnosis, which demonstrate the hematoma size and location of the hemorrhage. Standard CT can detect up to 95% of ICH; however patients presented several days after a small ICH may have little blood visible on a CT scan. MRI is known to overestimate the size of microbleed (the “blooming effect”), with MRI diameter on average more than 150% of pathological lesions [25, 26]. It is common for patients' condition to not allow MRI imaging to be done on a predetermined schedule, or earlier in the course of ICH. In addition, image techniques reflect the morphology rather than the function of the BBB. Under this circumstance, the availability of circulating biomarkers may assist in selection of those patients who require further investigation [27]. ICH results in the release of numerous blood/blood vessel broken products into the CSF, circulating through the ventricles, subarachnoid space, and parenchyma, and these substances have a long resistance time in CSF and CNS, making CSF be a choice for novel biomarker discovery [28–30]. Measurement of biomarkers in CSF may reflect accurately the status of ICH and serve as surrogate endpoints for experimental or nonoptimized therapies; some of these may be used in the early identification and diagnosis of the condition and its sequelae, as well as for determining the prognosis [31]. In this study, we assessed the changes of CLDN5, OCLN, and ZO-1 in both CSF and serum in patients with ICH to reveal whether circulating TJs provide a valuable predictor for the BBB disruption following ICH.

## 2. Method

### 2.1. Patients

This prospective study was approved by the Shantou University Medical College; written informed consents were obtained from the patients or their surrogates. Twenty-two patients were recruited during 2013-2014. Patients suspicious of ICH underwent immediately CT scanning or MRI after hospital admission. The diagnosis of ICH was confirmed by CT or MRI. The Digital Imaging and Communications in Medicine (DICOM) format was analyzed centrally for the measurement of hematoma size. As the CSF is very hard to get in health individuals, seventeen patients with disease other than ICH were enrolled as the control for comparison of the biomarkers in CSF. The details of control were shown in Table 1.

### 2.2. Hematoma Size Measurement

The size of the ipsilateral hemisphere and the infarcted area were measured using a standard computer-assisted analysis technique (ImageJ). Calculation of hematoma size was performed using the formula: size of infarcted area/ipsilateral hemisphere × 100 and shown as percentage of the whole hemisphere. The sizes were shown as mean ± SD.

### 2.3. Sample Collection and Measurement

Paired serum and CSF samples were obtained on patients admission. Lumbar puncture was used for diagnostic or treatment purposes. CSF sample was collected before treatment. Samples were immediately centrifuged, and supernatants were stored at −80°C until assessment. CSF cytology, total protein, and electrolyte were measured in all of the patients before therapy. The serum biomarkers were measured according to the same protocol as described for the CSF. VEGF and MMP-9 in CSF were measured by ELISA (R&D America Company). CSF and serum TJs levels were measured by ELISAs (ELISA kits from Cusabio (America) company).

### 2.4. Evaluation of BBB Integrity

The assessment of the BBB integrity in the patients and the controls was performed as previously described [19]. We explored whether the ratio of CLDN5, OCLN, and ZO-1 in CSF and serum reflects BBB disruption. We used the following formula:

(1) $$\begin{array}{c} \text{CLDN5 index}=\frac{{\text{CLDN5}}_{\text{CSF}}/{\text{CLDN5}}_{\text{Serum}}}{{Albumin}_{CSF}/{Albumin}_{Serum}}. \end{array}$$

Index of OCLN and ZO-1 was calculated with the same equation.

### 2.5. Statistical Analyses

Statistical analyses were performed using SPSS for Windows version 10.0. All continuous variables were reported as medians with interquartile ranges (IQR). Mann-Whitney *U* test, Spearman rank correlation, a multivariate regression analysis, and binary logistic regression were applied in the study. A cluster analysis (CA) and a principal component analysis (PCA) were performed to obtain significant principal components. *p* < 0.05 was considered statistically significant.

## 3. Results

### 3.1. General Characteristic of Patients with ICH and the Controls

Patient demographic and clinical data were summarized in Table 2. Of the 22 patients, the mean age of ICH group was 55.05 (range 49.18–60.91); 12 were men and 10 were women. Hypertension is the most common cause of ICH in our patients, accounting for all cases. The mean systolic blood pressure (SBP) was 175.5 (range: 157.0–190.5 mmHg), and diastolic blood pressure (DBP) was 104.5 (range: 94.5–111.25 mmHg) in ICH patients. SBP was significantly associated with the patient's primary outcome.

The WBC in CSF (WBC_CSF_) and blood were significantly higher in the ICH than in the controls (*p* < 0.05), indicating inflammatory status existed in ICH patients. We did not find significant differences in parameters (RBC, HB, and LDH in blood, serum enzyme, and glucose and Cl in CSF) between the ICH patients and the controls (*p* > 0.05). The ALB in CSF (ALB_CSF_) and BBB ratio in ICH group were significantly higher than in controls (*p* < 0.05); on the contrary, ALB_Serum_ in ICH was significantly lower than in controls (*p* > 0.05).

### 3.2. Serum and CSF CLDN5, OCLN, and ZO-1 Levels in Patients with ICH and the Controls

There were significantly higher levels of CLDN5, OCLN, and ZO-1 in CSF (CLDN5_CSF_, OCLN_CSF_, and ZO-1_CSF_) observed in ICH than in controls; specifically, CLDN5_CSF_ was 8.28-fold higher than that in control, OCLN_CSF_ was 18.78-fold higher than in control, and ZO-1_CSF_ was 5.16-fold higher than in control (*p* < 0.05). However, serum levels of CLDN5, OCLN, and ZO-1 had no difference between ICH group and control groups (*p* > 0.05). Our data revealed that circulating TJs increased in CSF but not in serum of patients suffering from ICH. We did not find significant differences of CLDN5/OCLN or CLDN5/ZO-1 index (both in serum and in CSF) between ICH and controls (*p* > 0.05) (Table 3).

### 3.3. Correlations among the Clinical Parameters and Circulating TJs Levels and BBB Malfunction in the ICH Patients

**Figure 1:**
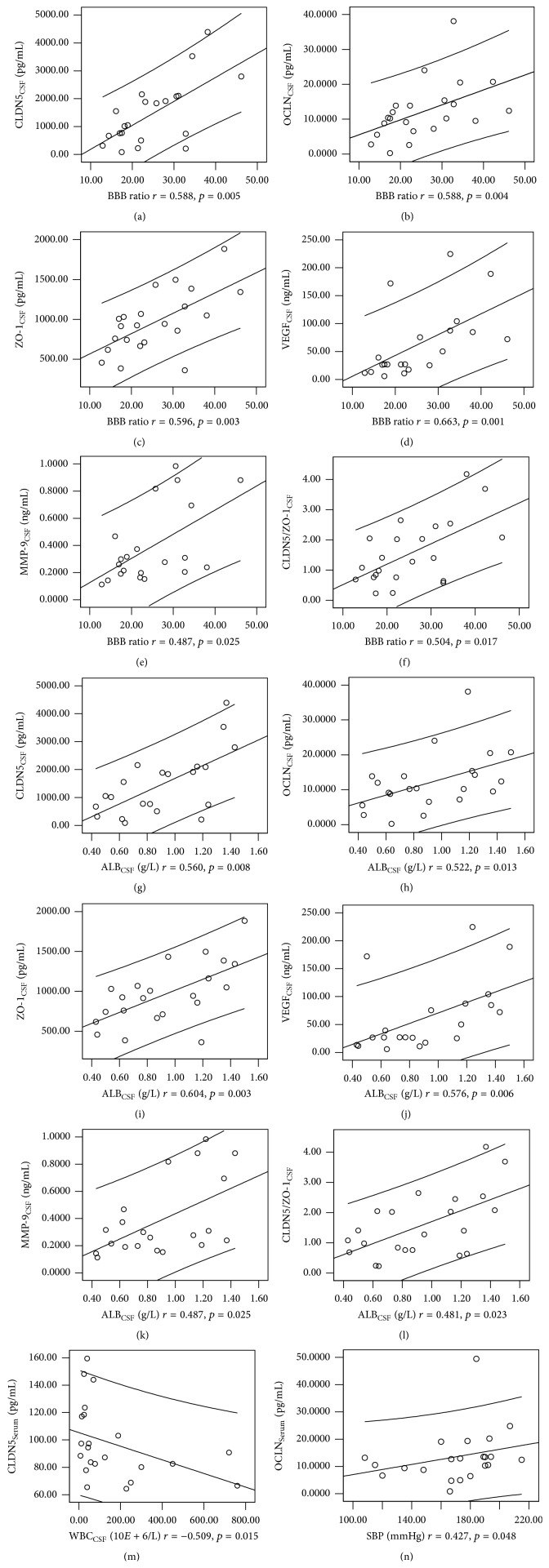
Correlations between parameters: positive correlations were found between BBB ratio and CLDN5_CSF_ (a), BBB ratio and OCLD_CSF_ (b), BBB ratio and ZO-1_CSF_ (c), BBB ratio and MMP-9_CSF_ (d), BBB ratio and VEGF_CSF_ (e), BBB ratio and CLDN5_CSF_/ZO-1_CSF_ (f), ALB_CSF_ and CLDN5_CSF_ (g), ALB_CSF_ and OCLD_CSF_ (h), ALB_CSF_ and ZO-1_CSF_ (i), ALB_CSF_ and MMP-9_CSF_ (j), ALB_CSF_ and VEGF_CSF_ (k), ALB_CSF_ and CLDN5_CSF_/ZO-1_CSF_ (l), and SBP and OCLN_Serum_ (n). Negative correlation was found between WBC_CSF_ and CLDN5_Serum_ (m).

A Spearman rank correlation was used to analyze the correlations among circulating CLDN5, OCLN, and ZO-1 and the conventional biomarkers known to be associated with the BBB integrity (BBB ratio, ALB_CSF_, and WBC_CSF_) (Table 4 and Figure 1). In the ICH group, the CLDN5_CSF_, OCLN_CSF_, and ZO-1_CSF_ were positively associated with ALB_CSF_ (*r* = 0.560, *p* = 0.008; *r* = 0.522, *p* = 0.013; and *r* = 0.604, *p* = 0.003, resp.) and BBB ratio (*r* = 0.588, *p* = 0.005; *r* = 0.588, *p* = 0.004; and *r* = 0.596, *p* = 0.003, resp.). No relationship was found between CSF TJs and WBC_CSF_ (*p* > 0.05), indicating that the inflammatory status may not affect the BBB integrity. The ratio of CLDN5/ZO-1 was positively associated with BBB ratio (*r* = 0.481, *p* = 0.023) and ALB_CSF_ (*r* = 0.504, *p* = 0.017). However, no relationship was found between serum TJs concentrations and the BBB conventional biomarkers (*p* > 0.05). In addition, no relationship was found between circulating TJs and area of hemorrhage (*p* > 0.05). Our data revealed that the efficiency of concentrations of CLDN5_CSF_, OCLN_CSF_, and ZO-1_CSF_ was higher than serum TJs and other conventional biomarkers in reflecting BBB integrity.

### 3.4. CSF VEGF and MMP-9 Levels in Patients with ICH and the Controls

We further analyzed the mechanism inducing CLDN5, OCLN, and ZO-1 increased in CSF that was observed in ICH group. VEGF_CSF_ and MMP-9_CSF_ were measured in ICH patients and the controls. Our data found that VEGF_CSF_ and MMP-9_CSF_ were significantly higher than those in control (*p* < 0.05). VEGF_CSF_ was positively associated with the BBB ratio (*r* = 0.663, *p* = 0.001), while MMP-9_CSF_ was positively related to BBB ratio and ALB_CSF_ (*r* = 0.487, *p* < 0.05) (Tables 3 and 4).

### 3.5. Diagnostic Significance of Circulating TJs in ICH

Diagnostic value of TJs levels in mirroring the BBB integrity was also evaluated. The sensitivities, specificities, positive predictive value (PPV), and negative predictive value (NPV) of circulating TJs in ICH diagnosis were shown in Table 5. Our data revealed that CSF levels of TJs had higher area under curve (AUC) and high sensitivity and specificity compared to the other conventional biomarkers studied. The sensitivity of CLDN5_CSF_ was 81.8%, and the specificity was 94.1%. The PPV of CLDN5_CSF_ was 94.74% and NPV was 80.0%. The sensitivities and specificities of OCLD_CSF_ and ZO-1_CSF_ were 95.5% versus 81.8% and 94.0% versus 94.0%, respectively. The PPV of OCLD_CSF_ and ZO-1_CSF_ were 95.5% versus 100% and NPV was 93.8% versus 85.0%. This trend seems to be not existing in the controls. On the contrary, serum levels of TJs had higher AUC and high sensitivity and specificity in control group when compared to the patients with ICH (data not shown). With respect to the VEGF_CSF_ and MMP-9_CSF_, they had high specificities and sensitivities. Collectively, our data showed that TJs in CSF have high sensitivity and specificity in diagnosis of ICH.

### 3.6. Principal Component Analysis (PCA) and Hierarchical Clustering of Candidate Parameters

**Figure 2:**
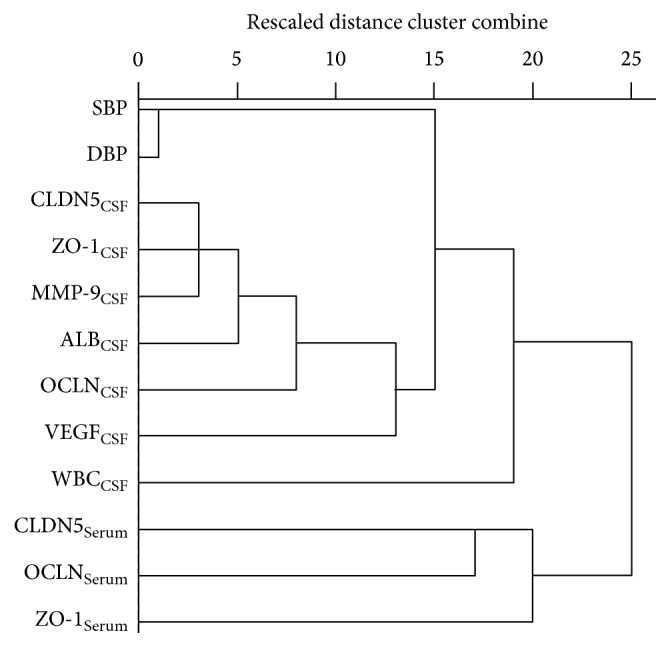
Dendrogram based on hierarchical agglomerative clustering of sampling stations using single linkage method. Parameters were classified into 3 main categories. The CLDN5_CSF_, ZO-1_CSF_, MMP-9_CSF_, ALB_CSF_, OCLN_CSF_, and VEGF_CSF_ were gathered together; the CLDN5_Serum_, OCLN_Serum_, and ZO-1_Serum_ were gathered together; the SBP and DBP were gathered together.

To identify a TJs profile that may provide a greater accuracy of class prediction than a single biomarker, we used the principal component analysis (PCA) (Figure 2) to choose a panel with the greatest accuracy of class prediction and the smallest misclassification error. PCA extracted four important principal components with eigenvalues >1, which explained 81.76% of total variance in the data set. The first model showed strong positive loadings (>0.75) for ALB_CSF_, CLDN5_CSF_, OCLD_CSF_, ZO-1_CSF_, and MMP-9_CSF_. The second model included OCLN_Serum_, ZO-1_Serum_, DBP, and SBP. The third model included WBC_CSF_ and DBP. However, the AUC value of Component 1 was 0.928, and the sensitivity was 81.8%, and the specificity was 94.1%, not higher than the single parameter from the overall level (Tables 6 and 7). To find out whether the difference is due to the small simple size, further study with larger sample size is needed.

## 4. Discussion

The need for new therapeutic approaches for ICH has prompted a search for the molecular and cellular mechanisms underlying early or delayed brain damage after ICH [32]. It is difficult to assess the importance of secondary BBB disruption following ICH that contributed to brain injury [33]. Blood/blood vessels breakdown products may be released in CSF following ICH, where CSF circulation plays a major role in clearing these products [34]. Therefore, the change of proteins in CSF is valuable in reflecting pathogenic status and prediction of ICH's outcome. In addition, circulating biomarkers are easily measurable, accurate, and cost-effective, making them more accessible in clinic. However, up to date, no circulating biomarkers mirror the degree of brain injury following ICH, nor have biomarkers predicting ICH's outcome been incorporated into routine clinical work [35].

In previous study, the observed BBB opening after SAH was related to the damage of tight junction [15]. The molecules released into the circulation occur during brain ischemia [21], indicating disassembly of TJs, and then disrupt the integrity of the BBB. Loss of ZO-1 and OCLN from cerebral vascular endothelium was also observed during CNS inflammation [36]. Supporting the point, we found that TJs_CSF_ were significantly higher in patients with ICH than the individuals who had no BBB damage. Together with a strong correlation existing between TJs concentration in CSF and the BBB ratio in patients with ICH, our data demonstrate the levels of TJs_CSF_ could be the potential biomarker for mirroring the extent of BBB opening in patients with ICH. In clinical practice, blood biomarkers are the first choice as many of them assist in rapid diagnosis, therapeutic decisions, and easier collection. Unfortunately, we could not find significant elevation of serum TJs nor correlation between CSF and serum's TJs. There may be two reasons to explain; the first one may be due to too small sample size in our study; statistical significance may be gained in a larger cohort. But there may be another reason: no correlation existed between CSF and serum. BBB breakdown is a local event of CNS; TJs elevated in CSF have difficulty inducing significant elevation of TJs in serum, especially considering the CSF volume versus serum volume. Further study is needed to elucidate the details. In conclusion, CSF biomarkers may have high sensitivity and specificity in reflecting CNS pathologic status, which is supported by our data that CSF levels of TJs had higher AUC and high sensitivity and specificity compared to serum TJs and other conventional serum biomarkers studied.

BBB disruption increases cerebrovascular permeability, allowing the entrance of leukocytes into the brain parenchyma which can in turn cause edema formation [9]. Higher WBC_CSF_ in ICH patients demonstrated that damaged BBB integrity leads to peripheral WBC transferred into the CNS. The TJs' completeness in BBB strictly depends on signals provided by the CNS microenvironment [37]. Increased permeability of the BBB can be caused by disruption of the extracellular matrix [38]. There is evidence that MMPs are increased after ICH [22] and involved in disruption of tight junctions, leading to increased BBB permeability and vasogenic brain edema [39]. Neutrophils are very important sources of MMP-9 after ICH [40]. In our study, significantly higher infiltrating leucocytes may contribute to higher level of MMP-9 in CSF after ICH, which should be a major source of MMP-9 in CSF [41]. MMP-9 degraded TJs and is associated with the disruption of OCLN and CLDN5 in brain endothelial cells [42–44]. Elevated MMP-9_CSF_ change the permeability of the BBB, participating in the opening of the BBB in ICH patients [45, 46]. Our data revealed that ICH induces BBB hyperpermeability through permeated neutrophils that released abundant MMP-9 in CSF, then, leading to TJs release into CSF.

Both BBB disruption and consequent vasogenic edema determine the clinical course of ICH [16, 47]. It is vital to understand the mechanisms underlying the mechanism of edema formation in ICH [33, 48]. VEGF is a factor that increases permeability of the endothelium [49]. Studies also show that VEGF disrupts TJs, induces breakdown of the BBB, and causes edema [50]. High permeability of blood vessel leads to leakage of large molecule and blood products into the vessel or perivascular space and induces serum proteins extravasated freely in the brain, forming vasogenic edema. In our study, VEGF_CSF_ in ICH patients is significantly higher than that in control, which is positively associated with the BBB ratio, revealing that higher concentration of VEGF_CSF_ contributes to the BBB permeability in ICH. VEGF perturbs TJ integrity by decreasing OCLN and ZO-1 expression and causing CLDN5 and ZO-1 protein disruption [50]. Positive associations were observed between BBB disruption and VEGF_CSF_, indicating that higher VEGF_CSF_ may be a crucial factor in BBB high permeability in ICH [51].

In some cases, the diameter of brain parenchyma blood vessels affected by ICH is too small to be observed by conventional imaging techniques. A recently proposed model of cerebral microscopic hemorrhage suggested that transient loss of endothelial barrier function might be an underlying process [26]. Hypertension, very high levels occurring in patients with ICH, can cause microaneurysms and small vessel wall damage and a gradual weakening of vascular integrity that eventually leads to rupture. Fluctuations in SBP as a result of impaired cerebral autoregulatory control in microvascular channels could promote hematoma expansion [52], but study also showed no association of blood pressure variability and hematoma growth [53], which is also observed in our study. Elevated blood pressure may cause endothelial dysfunction, leading to opening of the BBB [54]. To patients with microbleed or microvascular injury, circulating biomarker measurement may have some advantages in early diagnosis. Vascular damage induced by hypertension may be the initial cause of ICH and BBB dysfunction (with associated edema and leukocyte extravasation) is a secondary consequence [55].

Although early diagnosis and intervention may be paramount to improving patient prognosis [29], little is known about predictors for recurrence of primary ICH and particularly predictors for fatal recurrence [1]. MMP-9_CSF_, VEGF_CSF_, and TJs_CSF_ could be sensitive biomarkers as their expressions in some patients were upregulated just after ICH (data not shown). TJs have been considered attractive targets for transient breakdown of the BBB in therapies for various CNS disorders [13]. A panel of biomarkers may have far more discriminative power than any single biomarker alone to distinguish pathophysiological complications and brain damage following aSAH [31]. When we try to use the principal component analysis (PCA) to search a panel of biomarkers with the greatest accuracy of class prediction and the smallest misclassification error, the first model extracted was the ALB_CSF_, CLDN5_CSF_, OCLD_CSF_, ZO-1_CSF_, and MMP-9_CSF_, which could explain 81.76% of total variance in the data set. To find out whether the panel TJs in our study can reflect more accurately the BBB disruption and early brain injury after ICH than conventional biomarkers, larger sample size is needed.

The main limitation of TJs in CSF as the diagnostic biomarker is that LPs are not to be routinely done in some patients with ICH. Under this circumstance, circulating biomarkers and image detection should be carefully used according to patient's status. Another limitation of our study was that we could not measure the TJs at the different time points. Also, we have enrolled relatively small numbers of subjects that mainly have significantly large hematoma size. Our study will have better clinical significance if studying subject includes the patients with microbleeding; however, the patients' choice remains a challenge. Given that the CSF TJs serve diagnostic purposes for BBB disruption after ICH, further study will be needed to identify that the biomarkers could be translated into routine clinical practice. The sensitivity, specificity, PPV, and NPV for CSF TJs in diagnosis of ICH also need to be evaluated.

## Figures and Tables

**Table 1:** General character of the controls.

Controls	Gender	Age (yrs)	RBC_CSF_	WBC_CSF_	Glu_CSF_	Cl_CSF_	Alb_CSF_	BBB ratio	Diagnosis
1	M	12.00	1	11.00	3.66	128.10	0.40	9.69	CNS IRS
2	F	28.00	1	2.00	3.51	133.00	0.07	2.08	Headache
3	M	5.00	3	25.00	3.52	123.50	0.28	7.22	CNS IRS
4	M	28.00	0	1.00	3.26	128.60	0.49	13.17	Headache
5	M	2.50	0	4.00	3.45	122.70	0.12	3.13	Headache
6	M	50.00	0	38.00	3.46	108.00	1.01	23.82	Viral encephalitis
7	M	15.00	2	2.00	4.75	119.00	0.21	5.48	Headache
8	F	12.00	1	5.00	3.35	131.00	0.21	4.94	Suspected inflammation
9	M	43.00	0	49.00	3.73	124.90	0.47	13.28	Viral encephalitis
10	M	29.00	0	20.00	3.11	127.20	0.49	12.63	Viral encephalitis
11	M	15.00	20	65.00	4.32	128.70	0.37	8.71	Viral encephalitis
12	M	57.00	3	5.00	3.99	122.00	0.71	18.83	Suspected inflammation
13	M	5.00	1	5.00	3.20	124.80	0.33	8.51	Suspected inflammation
14	M	16.00	1	45.00	3.12	124.50	0.49	13.14	Viral encephalitis
15	F	50.00	4	5.00	5.00	129.00	0.48	11.19	Headache
16	M	35.00	2	48.00	3.89	134.80	0.80	20.41	Viral encephalitis
17	M	3.00	20	19.00	4.67	131.60	0.31	7.06	CNS IRS

M: male; F: female; CSF: cerebrospinal fluid; WBC_CSF_: white blood cell in CSF; RBC_CSF_: red blood cell in CSF; Alb_CSF_: albumin in CSF; Glu_CSF_: glucose in CSF; Cl_CSF_: chlorine in CSF; BBB ratio: Alb_CSF_ × 1000/Alb_Serum_; CNS IRS: CNS infection recovery stage.

**Table 2:** General characters of patients with intracranial hemorrhage and the controls.

	Intracranial hemorrhage	Controls
Age (yrs)	55.05 (49.18–60.91)	23.88 (14.70–33.06)
M/F	12/10	14/3
SBP (mmHg)	175.5 (157.00–190.50)^*∗∗*^	121.00 (100.50–133.00)
DBP (mmHg)	104.50 (94.50–111.25)^*∗∗*^	70.00 (66.50–82.00)
WBC_Blood_ (10*E* + 9/L)	11.94 (10.04–15.11)^*∗∗*^	7.61 (5.93–11.04)
RBC_Blood_ (10*E* + 12/L)	4.14 (3.80–4.48)	4.51 (4.27–4.75)
Hb_Blood_ (g/L)	123.95 (115.29–132.63)	128.06 (118.76–137.35)
PLT_Blood_ (10*E* + 9/L)	193.50 (143.00–246.00)	239.00 (169.00–313.00)
LDH_Serum_ (U/L)	247.14 (215.16–279.11)	201.29 (166.33–236.26)
AST_Serum_ (U/L)	47.50 (30.00–88.00)	26.00 (24.00–44.00)
ALT_Serum_ (U/L)	38.00 (19.00–90.00)^*∗*^	21.00 (15.00–33.00)
GGT_Serum_ (U/L)	52.50 (23.00–88.00)^*∗∗*^	17.00 (12.00–24.00)
ALP_Serum_ (U/L)	73.50 (56.00–87.00)	90.00 (54.00–154.00)
CHE_Serum_ (U/L)	5.85 (4.90–6.81)^*∗*^	7.16 (6.41–7.91)
TP_Serum_ (g/L)	65.71 (61.68–69.74)	67.50 (64.19–70.81)
ALB_Serum_ (g/L)	36.36 (34.09–38.63)^*∗*^	39.35 (37.90–40.81)
Glu_CSF_ (mmol/L)	3.93 (2.98–4.26)	3.52 (3.31–4.16)
Cl_CSF_ (mmol/L)	121.17 (118.68–123.65)^*∗*^	125.96 (122.75–129.17)
ALB_CSF_ (g/L)	0.93 (0.78–1.08)^*∗∗*^	0.43 (0.30–0.55)
WBC_CSF_ (10*E* + 6/L)	52.50 (28.00–233.50)^*∗∗*^	11.00 (4.50–41.50)
BBB ratio	22.68 (17.46–32.79)^*∗∗*^	9.69 (6.27–13.23)

M: male; F: female; SBP: systolic blood pressure; DBP: diastolic blood pressure; CSF: cerebrospinal fluid; WBC: white blood cell; RBC: red blood cell; Hb: hemoglobin; PLT: platelet; LDH: L-lactate dehydrogenase; AST: aspartate aminotransferase; ALT: alanine aminotransferase; GGT: gamma-glutamyl transpeptidase; ALP: alkaline phosphatase; CHE: cholinesterase; TP: total protein; Alb: albumin; GLB: globulin; Glu: glucose; Cl: chlorine; BBB ratio: Alb_CSF_ × 1000/Alb_Serum_.

^*∗*^ *p* ≤ 0.05 and ^*∗∗*^ *p* ≤ 0.01 when the intracranial hemorrhage group is compared with control.

**Table 3:** Circulating TJs and cytokines in patients with intracranial hemorrhage and the controls.

	Intracranial hemorrhage	Controls
CLDN5_CSF_ (pg/mL)	1302.35 (669.72–2103.04)^*∗∗*^	157.28 (107.48–213.82)
CLDN5_Serum_ (pg/mL)	89.62 (80.18–117.07)^*∗∗*^	125.79 (103.07–125.79)
OCLN_CSF_ (pg/mL)	10.30 (7.22–14.26)^*∗∗*^	0.55 (0.39–1.48)
OCLN_Serum_ (pg/mL)	12.56 (8.75–13.54)	10.95 (9.63–10.95)
ZO-1_CSF_ (pg/mL)	934.46 (712.03–1161.35)^*∗∗*^	181.08 (138.46–214.08)
ZO-1_Serum_ (pg/mL)	2007.96 (1506.78–2647.24)^*∗∗*^	2173.48 (1678.81–2173.48)
CLDN5_CSF_/OCLN_CSF_	145.55 (75.62–225.66)	268.65 (116.65–452.29)
CLDN5_Serum_/OCLN_Serum_	7.77 (6.24–12.02)	11.48 (10.70–13.26)
CLDN5_CSF_/ZO-1_CSF_	1.34 (0.76–2.08)	0.93 (0.57–1.39)
CLDN5_Serum_/ZO-1_Serum_	0.0489 (0.0413–0.0587)	0.0579 (0.0403–0.0579)
VEGF_CSF_ (ng/mL)	33.16 (25.42–87.43)^*∗∗*^	9.21 (8.38–10.03)
MMP-9_CSF_ (ng/mL)	0.29 (0.20–0.69)^*∗∗*^	0.10 (0.09–0.13)

CLDN5_Serum_: serum Claudin-5; OCLN_Serum_: serum Occludin; ZO-1_Serum_: serum ZO-1; CSF: cerebrospinal fluid; CLDN5_CSF_: Claudin-5 in CSF; OCLN_CSF_: OCLN in CSF; ZO-1_CSF_: ZO-1 in CSF; VEGF: vascular endothelial growth factor; MMP-9: metalloproteinases.

^*∗∗*^ *p* ≤ 0.01 when the intracranial hemorrhage group is compared with control.

**Table 4:** Correlations between TJs and other biomarkers in intracranial hemorrhage group.

	CLDN5_CSF_	CLDN5_Serum_	OCLN_CSF_	OCLN_Serum_	ZO-1_CSF_	ZO-1_Serum_	VEGF_CSF_	MMP-9_CSF_	CLDN5_CSF_/ZO-1_CSF_	CLDN5_Serum_/ZO-1_Serum_	Area of hemorrhage
SBP	*r* = 0.402	*r* = 0.150	*r* = 0.024	*r* = 0.427	*r* = 0.067	*r* = 0.188	*r* = 0.097	*r* = 0.047	*r* = 0.242	*r* = −0.204	*r* = −0.130
	*p* = 0.071	*p* = 0.504	*p* = 0.915	*p* = 0.048^*∗*^	*p* = 0.766	*p* = 0.402	*p* = 0.675	*p* = 0.838	*p* = 0.277	*p* = 0.363	*p* = 0.619

DBP	*r* = 0.365	*r* = −0.037	*r* = 0.015	*r* = 0.258	*r* = 0.197	*r* = 0.100	*r* = −0.009	*r* = 0.177	*r* = 0.139	*r* = −0.219	*r* = −0.332
	*p* = 0.104	*p* = 0.871	*p* = 0.948	*p* = 0.246	*p* = 0.380	*p* = 0.657	*p* = 0.969	*p* = 0.444	*p* = 0.537	*p* = 0.327	*p* = 0.193

Alb_CSF_	*r* = 0.560	*r* = −0.132	*r* = 0.522	*r* = 0.001	*r* = 0.604	*r* = 0.053	*r* = 0.576	*r* = 0.487	*r* = 0.481	*r* = 0.003	*r* = 0.191
	*p* = 0.008^*∗∗*^	*p* = 0.558	*p* = 0.013^*∗*^	*p* = 0.996	*p* = 0.003^*∗∗*^	*p* = 0.814	*p* = 0.006	*p* = 0.025^*∗*^	*p* = 0.023^*∗*^	*p* = 0.988	*p* = 0.462

WBC_CSF_	*r* = −0.204	*r* = −0.509	*r* = −0.293	*r* = −0.089	*r* = −0.115	*r* = −0.292	*r* = −0.021	*r* = 0.136	*r* = −0.121	*r* = −0.125	*r* = −0.134
	*p* = 0.375	*p* = 0.015^*∗*^	*p* = 0.185	*p* = 0.694	*p* = 0.610	*p* = 0.188	*p* = 0.926	*p* = 0.557	*p* = 0.592	*p* = 0.580	*p* = 0.609

BBB ratio	*r* = 0.588	*r* = −0.167	*r* = 0.588	*r* = 0.013	*r* = 0.596	*r* = 0.062	*r* = 0.663	*r* = 0.487	*r* = 0.504	*r* = −0.107	*r* = 0.265
	*p* = 0.005^*∗∗*^	*p* = 0.459	*p* = 0.004^*∗∗*^	*p* = 0.954	*p* = 0.003^*∗∗*^	*p* = 0.784	*p* = 0.001^*∗∗*^	*p* = 0.025^*∗*^	*p* = 0.017^*∗*^	*p* = 0.636	*p* = 0.305

Area of hemorrhage	*r* = 0.471	*r* = 0.147	*r* = 0.362	*r* = −0.307	*r* = 0.270	*r* = 0.338	*r* = 0.277	*r* = 0.219	*r* = 0.407	*r* = −0.210	
	*p* = 0.066	*p* = 0.573	*p* = 0.153	*p* = 0.231	*p* = 0.295	*p* = 0.184	*p* = 0.300	*p* = 0.415	*p* = 0.105	*p* = 0.419	

CLDN5_Serum_: serum Claudin-5; OCLN_Serum_: serum Occludin; ZO-1_Serum_: serum ZO-1; CSF: cerebrospinal fluid; CLDN5_CSF_: Claudin-5 in CSF; OCLN_CSF_: OCLN in CSF; ZO-1_CSF_: ZO-1 in CSF; VEGF: vascular endothelial growth factor; MMP-9: metalloproteinases.

SBP: systolic blood pressure; DBP: diastolic blood pressure.

^*∗*^ *p* ≤ 0.05 and ^*∗∗*^ *p* ≤ 0.01 when the intracranial hemorrhage group is compared with control.

**Table 5:** Receiver operating characteristic curve analysis of the intracranial hemorrhage group.

	AUC	*p*	Cut-off value	Sensitivity	Specificity	PPV	NPV
ZO-1_CSF_ (pg/mL)	0.992	0.000	374.21	95.50	94.10	95.50	94.10
OCLN_CSF_ (pg/mL)	0.960	0.000	2.19	95.50	94.10	95.50	94.10
CLDN5_CSF_ (pg/mL)	0.930	0.000	409.18	81.80	94.10	94.74	80.00
MMP-9_CSF_ (ng/mL)	0.930	0.000	0.19	81.80	94.10	94.70	80.00
SBP (mmHg)	0.898	0.000	159.50	77.30	94.10	94.70	80.00
ALB_CSF_ (g/L)	0.896	0.000	0.50	90.90	82.40	87.00	87.50
DBP (mmHg)	0.893	0.000	95.00	77.30	94.10	94.40	76.20
VEGF_CSF_ (ng/mL)	0.869	0.000	11.28	90.90	82.40	87.00	87.50
WBC_CSF_ (10*E* + 6/L)	0.837	0.000	22.50	86.40	64.70	76.00	78.60
OCLN_Serum_ (pg/mL)	0.567	0.479	11.72	54.50	88.20	86.70	62.50
ZO-1_Serum_ (pg/mL)	0.432	0.470	2344.28	36.40	82.40	72.70	50.00
CLDN5_Serum_ (pg/mL)	0.251	0.008	68.25	86.40	11.80	90.50	83.30

AUC: areas under curve; PPV: positive predictive value; NPV: negative predictive value; CLDN5_Serum_: serum Claudin-5; OCLN_Serum_: serum Occludin; ZO-1_Serum_: serum ZO-1; CSF: cerebrospinal fluid; CLDN5_CSF_: Claudin-5 in CSF; OCLN_CSF_: OCLN in CSF; ZO-1_CSF_: ZO-1 in CSF; VEGF: vascular endothelial growth factor; MMP-9: metalloproteinases; SBP: systolic blood pressure; DBP: diastolic blood pressure.

**Table 6:** Loading scores of variables on the first three significant principal components.

	Component 1	Component 2	Component 3	Component 4
ZO-1_CSF_ (pg/mL)	**0.882 **	0.041	0.136	0.058
MMP-9_CSF_ (ng/mL)	**0.869 **	−0.104	0.189	0.185
CLDN5_CSF_ (pg/mL)	**0.847 **	0.114	0.090	0.181
ALB_CSF_ (g/L)	**0.750 **	0.086	0.464	−0.017
VEGF_CSF_ (ng/mL)	**0.582 **	−0.307	−0.178	−0.224
OCLN_CSF_ (pg/mL)	**0.457 **	−0.150	0.383	**−0.565 **
ZO-1_Serum_ (pg/mL)	0.356	**0.506 **	**−0.503 **	0.028
CLDN5_Serum_ (pg/mL)	0.355	0.278	**−0.801 **	−0.061
OCLN_Serum_ (pg/mL)	0.279	**0.690 **	−0.238	0.309
WBC_CSF_ (10*E* + 6/L)	−0.184	−0.005	**0.458 **	**0.743 **
DBP (mmHg)	−0.204	**0.756 **	**0.469 **	−0.194
SBP (mmHg)	−0.221	**0.803 **	0.318	−0.269

Bold values indicate strong and moderate loadings: Component 1 has strong positive loadings on ZO-1_CSF_, MMP-9_CSF_, CLDN5_CSF_, Alb_CSF_, VEGF_CSF_, and OCLN_CSF_; Component 2 has positive loadings on ZO-1_Serum_, OCLN_Serum_, DBP, and SBP; Component 3 has positive loadings on WBC_CSF_, DBP and negative loadings on ZO-1_Serum_, CLDN5_Serum_; Component 4 has positive loadings on WBC_CSF_ and negative loadings on OCLN_CSF_.

**Table 7:** Receiver operating characteristic curve analysis of the components.

	AUC	*p*	Cut-off value	Sensitivity	Specificity	PPV	NPV
Component 1	0.928	0.000	−0.708	81.8	94.1	94.7	80.0
Component 2	0.794	0.002	−0.559	72.7	88.2	88.2	68.2
Component 3	0.939	0.000	−1.005	86.4	94.1	95.0	84.2
Component 4	0.317	0.041	0.827	22.7	94.1	85.7	50.0

AUC: areas under curve; PPV: positive predictive value; NPV: negative predictive value.
